# Isolated attosecond pulse generation in a semi-infinite gas cell driven by time-gated phase matching

**DOI:** 10.1038/s41377-024-01564-5

**Published:** 2024-08-20

**Authors:** Federico Vismarra, Marina Fernández-Galán, Daniele Mocci, Lorenzo Colaizzi, Víctor Wilfried Segundo, Roberto Boyero-García, Javier Serrano, Enrique Conejero-Jarque, Marta Pini, Lorenzo Mai, Yingxuan Wu, Hans Jakob Wörner, Elisa Appi, Cord L. Arnold, Maurizio Reduzzi, Matteo Lucchini, Julio San Román, Mauro Nisoli, Carlos Hernández-García, Rocío Borrego-Varillas

**Affiliations:** 1 Department of Physics, Politecnico di Milano, Piazza Leonardo da Vinci 32, 20133 Milano, Italy; 2 IFN-CNR, Piazza Leonardo da Vinci 32, 20133 Milano, Italy; 3 Grupo de Investigación en Aplicaciones del Láser y Fotónica, Departamento de Física Aplicada, Universidad de Salamanca, E-37008 Salamanca, Spain; 4 Unidad de Excelencia en Luz y Materia Estructuradas (LUMES), Universidad de Salamanca, Salamanca, Spain; 5 Laboratorium für Physikalische Chemie, ETH Zürich, 8093 Zürich, Switzerland; 6 Department of Physics, Lund University, Lund, Sweden

**Keywords:** High-harmonic generation, Nonlinear optics, Ultrafast lasers

## Abstract

Isolated attosecond pulse (IAP) generation usually involves the use of short-medium gas cells operated at high pressures. In contrast, long-medium schemes at low pressures are commonly perceived as inherently unsuitable for IAP generation due to the nonlinear phenomena that challenge favourable phase-matching conditions. Here we provide clear experimental evidence on the generation of isolated extreme-ultraviolet attosecond pulses in a semi-infinite gas cell, demonstrating the use of extended-medium geometries for effective production of IAPs. To gain a deeper understanding we develop a simulation method for high-order harmonic generation (HHG), which combines nonlinear propagation with macroscopic HHG solving the 3D time-dependent Schrödinger equation at the single-atom level. Our simulations reveal that the nonlinear spatio-temporal reshaping of the driving field, observed in the experiment as a bright plasma channel, acts as a self-regulating mechanism boosting the phase-matching conditions for the generation of IAPs.

## Introduction

Since its initial demonstration^1^, the generation and manipulation of isolated attosecond pulses (IAPs) have provided groundbreaking tools for tracking electronic motion in atoms, molecules, and solids^2^. As a result, several fundamental processes, such as photoionization time delays^3^, charge migration^4^, electronic decoherences^5^, or carrier motion in solids^6^, have been observed and measured with unparalleled time resolution, opening new and exciting fields of research.

In conventional high-order harmonics generation (HHG) table-top setups, an intense (~10^14^–10^15^ W·cm^−2^) femtosecond driving laser field, centred in the near-infrared (IR) spectral region, is focused on a noble gas target. When macroscopic phase matching in the gas is ensured, the highly non-linear response of the medium leads to the coherent production of extreme-ultraviolet (XUV) radiation^7,8^. The natural form of this radiation is a train of attosecond pulses separated by half an optical cycle of the driving field^9^. The isolation of a single attosecond pulse from this train relies either on the use of extremely short driving pulses^10^ or on specific techniques, known as gating techniques^11^. For instance, polarization gating^12,13^ relies on a time-dependent polarization shaping of the driving radiation. Colour gating^14^, instead, introduces a second colour to break the electric field symmetry. A combined scheme that integrates both polarization and colour gating, the double optical gating approach^15^, can also be followed. Another widespread technique is ionization gating^16,17^, which utilizes a time-gated window for HHG phase matching^18^.

HHG efficiency is largely affected by the phase-matching of the harmonic radiation from all emitters in the generating medium^19–21^. Recent theoretical efforts^22^, later confirmed experimentally for trains of attosecond pulses^23^, have demonstrated two equivalent regimes of harmonic phase matching for efficient HHG, namely high-pressure in short-medium, with length *L* smaller than the laser Rayleigh range, *z*_*R*_ (*L* *<<* *z*_*R*_), and low gas pressure in long medium with *L* ≈ *z*_*R*_. It is important to note that the term “long medium” refers to the ratio of the medium length with respect to the Rayleigh length and it is not necessarily related to extended geometries (i.e., an HHG scheme for which the focusing length is typically >1 m and the energy of the driving pulse is greater than a few mJ). This latter scheme is prevailing in high-flux setups, such as the beamlines at NEXUS, SYLOS GHHG long beamline in ELI-ALPS^23^, Lund^23^ and MBI^24^. Currently, most laboratories utilize short-medium gas cells operated at high gas pressures for IAP generation. However, when scaling up the driving pulse energy becomes necessary, a long-medium scheme is generally more feasible than a short-medium one^23^. At the same time, long medium schemes operating at low pressure can lead to similar, if not higher, conversion efficiencies than static cells and pulsed valves^25^ and generally provide better spatiotemporal properties of the generated XUV radiation^22,23^. Moreover, prior research^26^ has emphasized the beneficial impact of terminating any generation process with a sharp pressure gradient. This approach generally promotes phase matching and restricts re-absorption, enhancing HHG efficiency. Despite these potential advantages, the possibility of generating IAPs in a long medium, such as semi-infinite gas cells (SIGCs)^27–29^, has not been fully established yet. On the contrary, due to the nonlinear phenomena that take place in a long medium and challenge favourable phase-matching conditions, these sources are commonly perceived to be inherently unsuited for IAP generation. Additionally, to model this regime, more advanced simulation tools are needed to support the interpretation of experimental results. These tools should be capable of simultaneously addressing the intricate nonlinear spatiotemporal dynamics of the driving radiation as it traverses the gas medium and the subsequent generation, propagation, and phase matching of harmonics. Consequently, due to the intrinsic complexity of modelling these processes, the mechanisms that could lead to IAPs generation in extended gas media have not been thoroughly investigated.

In this work, we present clear experimental evidence of efficient generation of IAPs in a SIGC geometry, unveiling the phase-matching mechanism in this regime (i.e., *L* *»* *z*_*R*_ and peak powers well below the critical power for filamentation). Our theoretical simulations show that the nonlinear spatiotemporal reshaping of the driving field in the SIGC acts as a self-regulating mechanism boosting the IAP generation, thanks to favourable phase-matching conditions. By reporting their adoption in an attosecond streaking measurement we not only demonstrate their suitability for real attosecond pump-probe experiments but also provide a reliable temporal characterization, revealing a duration of 180 as in the 20–45 eV spectral region. To understand the relevant role of harmonic phase-matching, we develop a novel 3D simulation method for macroscopic HHG, which combines nonlinear spatiotemporal reshaping of an intense driving few-cycle pulse with the solution of the three dimensional time-dependent Schrödinger equation (3D-TDSE) to account for the quantum dynamics of microscopic HHG. This approach allows us to unravel the role of the spatiotemporal nonlinear reshaping of the driving field as it propagates through the SIGC, as well as to identify a pressure-dependent phase-matching window that enables efficient generation of IAPs.

## Results

**Fig. 1:**
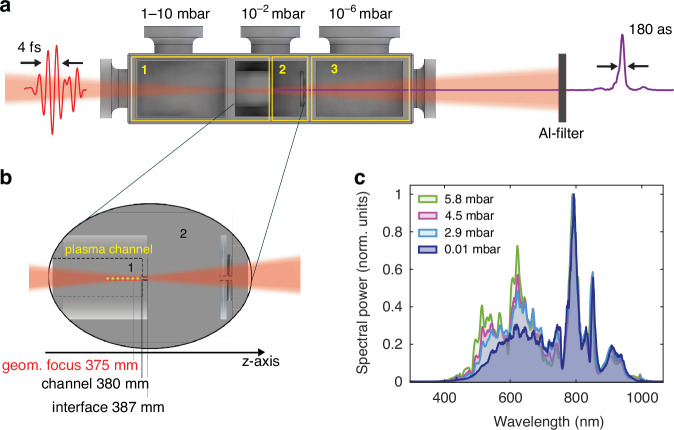
Experimental setup. **a** Scheme of the SIGC: the IR driving field, characterized by a streaking measurement, focuses after 375 mm, 5 mm before the cell-exit channel (at 380 mm). The first region (1) is filled with Argon gas (constant pressure tuneable from <1 mbar up to 10 mbar). A second chamber (2), which is maintained at 0.01 mbar vacuum using a roughing pump, separates (1) from a high vacuum region (3) (10^−6^ mbar) achieved by a turbo-pump, allowing easy interfacing of the SIGC with the rest of the beamline. Output XUV pulses with a duration of 180 as are characterized by a streaking measurement. **b** Detail of the region around chamber (1) and (2). A 7 mm channel connects the first chamber with the intermediate one (2). In the region around the geometrical focus *z* = 375 mm, as highlighted in the figure, a bright plasma channel appears for pressure >2 mbar. **c** Spectra of the IR driving field measured at the output of the SICG for various pressure configurations, as indicated in the legend

The SIGC consists of three main regions^30^, as shown in Fig. 1a. In the first region, the IR pulse interacts with a noble gas (Argon) and generates high-order harmonics in the XUV (20–45 eV). In this section, the pressure is homogeneously distributed and can be tuned within a range of up to 10 mbar. The gas region extends into the second chamber, through a mechanically adjustable element that allows for tuning the length of the gas-filled region from 370 mm to 400 mm. As shown in Fig. 1b, following this component, a plate with a drilled hole of 200 *µ*m diameter and 7 mm length connects the first chamber to the second one, where a roughing pump stabilizes the pressure at 0.01 mbar. In our geometry, the SIGC length is set at 380 mm and the IR focus lies 5 mm before the exit hole. A second plate with a 500 *µ*m diameter hole separates the second chamber from the last one, where the pressure is kept at 10^−6^ mbar using a turbo pump with an effective speed of 80 l·s^−1^. At increasing values of pressure in the first chamber, we observed the gradual development of a bright plasma channel extending over a few centimetres. Consequently, as depicted in Fig. 1c, we conducted a comprehensive characterization of the IR spectrum after generation at different operating pressures using a standard spectrometer (AvaSpecULS4096).

**Fig. 2:**
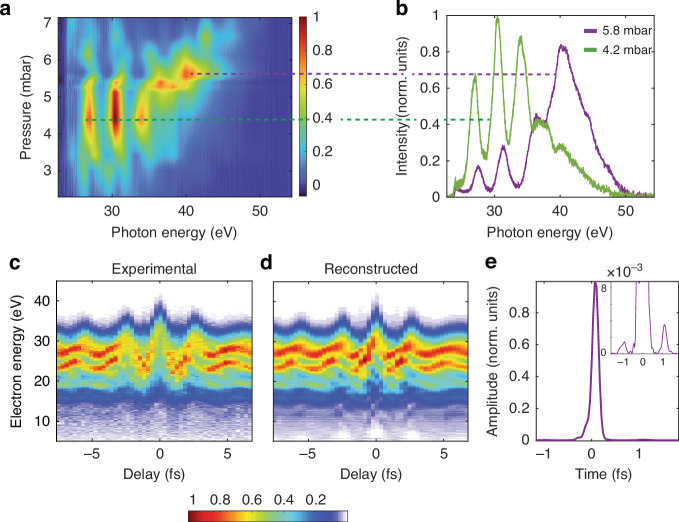
Spectral and temporal characterization of IAP generated in a SIGC. **a** Evolution of the spectrum of the XUV pulses as a function of the gas pressure. Around 5.5 mbar the spectrum becomes more continuous. **b** Spectra for two values of pressures in (**a**), 4.2 mbar (green curve) and 5.8 mbar (purple curve), respectively. **c** Experimental streaking trace in the case of 5.8 mbar. **d** Reconstructed streaking spectrogram with the ePIE algorithm. **e** Reconstructed XUV intensity profile, showing a clean IAP with a duration of 180 as. In the insert, <1% IAP satellites are shown; these structures are compatible with the modulation observed in both the trace reported in (**c**) and the violet XUV spectrum in (**b**)

As presented in Fig. 2a, we have measured the evolution of the HHG spectra upon varying the gas pressure in the SIGC. For each value of the pressure, the CEP of the driving IR pulses was optimized for the generation of continuous XUV spectra at high photon energy (above ≈ 35 eV). The results indicate that the high-order harmonics are phase-matched within a narrow pressure range between 3 and 7 mbar. Notably, we have observed that the recorded harmonic spectrum remains relatively stable against slight variations in the 200 *µ*m exit pinhole longitudinal position (±5 mm from the reported configuration).

As the pressure is increased, a transition of the XUV spectrum from modulated (green curve in Fig. 2b, at 4.2 mbar) to a quasi-continuum (violet curve in Fig. 2b, at 5.8 mbar) can be observed. In addition, the latter is accompanied by a noticeable reduction of the lower-order harmonics, as well as by an enhancement of the higher-order ones, close to the cutoff frequency. This continuum spectrum is sensitive to CEP variations (see Supplementary Material), differently from what has been observed for short media at similar intensities of the driving field^1,31^. Importantly, this shift from discrete to a quasi-continuum spectrum when increasing the pressure occurs without observing significant change in the overall harmonic flux. The conversion efficiency is estimated to be ~10^−6^, higher than what reported for the polarization gating technique^12,13^ and similar to what was obtained with the ionization gating^16^ in a 3 mm static cell using the same beamline. This aspect presents a distinct advantage compared to other gating methods (e.g. polarization gating) where a continuum spectrum is achieved at the expense of XUV flux.

We have then measured the temporal characteristics of the XUV pulses generated at 5.8 mbar, using the Frequency-Resolved Optical Gating for Complete Reconstruction of Attosecond Bursts (FROG CRAB), or simply streaking technique^32^. A portion of the residual IR radiation, not involved in the generation process, is delayed relative to the XUV pulse, and focused (I_*peak*_ = 5 × 10^12^ W·cm^−2^) in the TOF spectrometer where an Argon target is present. The resulting interaction leads to a streaking spectrogram, as demonstrated in Fig. 2c. To confirm the presence of an IAP, we performed a numerical reconstruction of the trace using the extended ptychographic iterative engine (ePIE)^33^. The reconstructed trace, illustrated in Fig. 2d, allowed us to retrieve both the IR field and the XUV pulse intensity. As shown in Fig. 2e, the ≈6 mbar configuration indeed supports an IAP with an intensity full-width at half maximum (FWHM) duration of 180 as. Furthermore, we can observe the presence of *<* 1% satellites (in intensity) of the IAP. These features are responsible for the residual modulations observed in both the photoelectron and XUV spectra. However, for most spectroscopic applications requiring IAPs, these temporal structures can be considered irrelevant. To gain a deeper insight into the physical mechanism behind the IAP generation in the SIGC, we have developed a novel theoretical approach that combines nonlinear IR propagation with macroscopic HHG, as described in the Methods section.

**Fig. 3:**
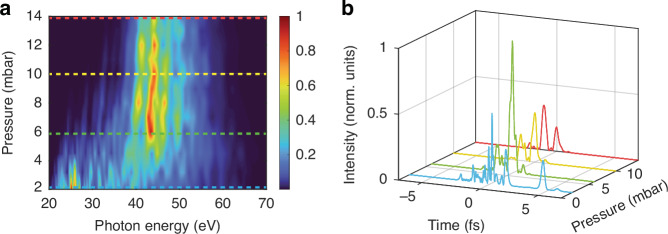
Simulated XUV pulses as a function of the gas pressure. **a** Simulation results of the XUV spectrum as a function of the gas pressure. **b** On-axis IAP obtained at pressure values of 2 mbar (blue), 6 mbar (green), 10 mbar (orange), and 14 mbar (dark red) after performing the Fourier transform of a sub-set of the HHG spectra indicated by dotted lines in (**a**)

In Fig. 3, we show the simulation results for the generation of IAPs in the SIGC. The propagated IR electric field in the cell is used as an input for macroscopic HHG calculations through 3D-TDSE and propagation of the harmonics. This allows us to compute the spatially averaged HHG emission spectrum at the far field detector by integrating along the azimuthal coordinate and the divergence angle up to 1.5 mrad, where most of the XUV radiation is emitted. The radiation was also propagated through a 150 nm thick Al filter (including both material absorption and dispersion), and a response function was assumed for the XUV camera to mimic the experimental measurements (high-pass filter from ≈23 eV). As depicted in Fig. 3a, the computed harmonic spectra align with the trends found in the experiment, despite a slight deviation between the experimental and theoretical pressure range of phase matching and the cleanness of the IAP. As the pressure is increased, the HHG spectrum is shifted towards higher energies, and becomes less modulated, thus favouring the generation of IAPs. This becomes evident also when comparing the time evolution of the radiation on axis in Fig. 3b. As we increase the pressure, the temporal profile of the XUV radiation becomes less modulated, with the cleanest temporal structure achieved at 6 mbar. Furthermore, the simulation is in general agreement with the experimental trend, which demonstrates a gradual decrease in generation efficiency as the pressure increases.

As illustrated experimentally and theoretically in Figs. 2 and 3, two crucial features in our HHG spectrum support the generation of an IAP: the formation of a continuous cut-off and the re-absorption of low-order harmonic components. In the following, we aim to address the origin of these features.

## Discussion

In order to understand the physics behind the IAP mechanism, we investigate the effects of the nonlinear propagation of the driving pulse. Several works in literature have reported that nonlinear effects can lead to the generation of an IAP. In ref.^34^ Steingrube and co-workers investigated the formation of continuous XUV spectra emerging from a filament by employing a loose focusing geometry (*L/z*_*R*_ ~14.2) at moderate intensities (*I*_*0*_ = 8 × 10^13 ^ W·cm^−2^ in vacuum) and high pressures (1 bar)^34^. A theoretical work by Gaarde and Schafer^31^ (related to the experiments presented in^1^) and the experiments by Haworth and co-workers^35^ discussed instead nonlinear propagation in short media (*L/z*_*R*_ < 0.15) at moderate pressures (few hundreds of mbar) and high intensities (between 6 and 9 × 10^14^ W·cm^−2^), showing that defocusing of the driving field leads to on-axis IAP generation. All these studies have thus addressed the effect of nonlinearities in IAP generation either in long media (*L»z*_*R*_) at peak powers above the critical power for self-focusing^34^ (*P*_c_) or short media (*L* < *z*_*R*_) at low peak powers^1,16,17,35^ (*P* « *P*_c_). Here we deal with a different scenario: long medium geometries (*L/z*_*R*_ ~15.1) at low pressures (6 mbar) and high intensities (*I*_*0*_ = 1.2 × 10^15^ W·cm^−2^ in vacuum), exploring therefore long media (*L* *»* *z*_*R*_) at peak powers well below the threshold for self-focusing.

**Fig. 4:**
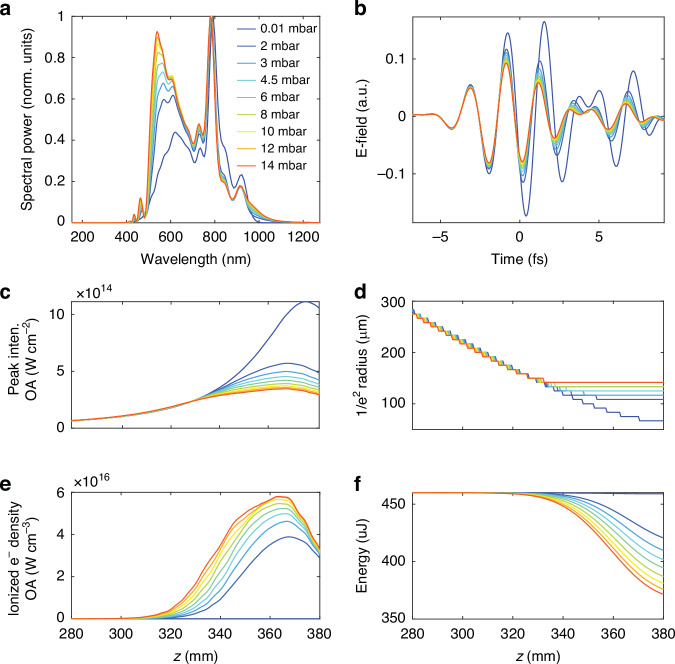
IR nonlinear propagation. **a** IR spectra and (**b**) corresponding on-axis electric field obtained at the SIGC output with the nonlinear propagation code (utilizing the experimental parameters as input) as a function of the gas pressure in the first chamber. **c**–**f** Simulated nonlinear reshaping of the driving field as it propagates along the *z*-direction in the final portion of the cell around the geometrical focus: (**c**) on-axis peak intensity, (**d**) beam radius, (**e**) on-axis ionized electron density and (**f**) pulse energy during propagation under different pressure conditions

To validate our numerical methodology, we start by examining the experimental IR spectra measured at the output of the SIGC when driven at varying pressures. As shown in Fig. 1c, the experimental data reveals a distinct ionization-induced self-phase modulation effect, which manifests as a clear blue shift of the spectrum. This process has been noted in recent works to enhance generation efficiency^36,37^. Figure 4a shows the simulated IR spectra after nonlinear propagation in the SIGC for different Ar pressures, accurately reproducing the experimental blue shift (Fig. 1c). Figure 4b depicts the corresponding reshaping of the IR driving pulse at the output of the SIGC.

With this empirical validation, we proceed to numerically monitor the nonlinear modification of the driving field as it propagates along the *z*-direction in the cell. This allows for a deeper understanding of the spatiotemporal reshaping of the IR driving radiation along the cell for different pressures. To this end, we analyse the evolution of the on-axis peak intensity (Fig. 4c), the beam radius (Fig. 4d), the ionized population (Fig. 4e), and the pulse energy (Fig. 4f) under different pressure conditions. Noticeably, as the gas pressure within the SIGC increases, we note a progressive beam size stagnation (Fig. 4d) and a reduction in the pulse energy (Fig. 4f), attributed, respectively, to plasma defocusing and losses due to ionization and plasma absorption. These phenomena collectively lead to an overall reduction in the peak intensity (Fig. 4c) of the driving field, as compared to linear propagation in vacuum. The observed reduction in peak intensity due to plasma defocusing and absorption appears to act as a self-regulating mechanism, which has been recently shown to contribute to efficient harmonic generation^36,38^. We should note that the measured HHG cutoff energy corresponding to the decreased intensity is in good agreement with the classical cutoff law, deviating from the much higher value that would be expected from the peak intensity of the beam focused in vacuum. This regulating mechanism also leads to a region of space where the driving field intensity and phase remain nearly constant along the propagation direction in the final part of the SIGC, which is the most relevant one for HHG as it is less affected by harmonic reabsorption. This peculiar condition, mostly induced by plasma de-focusing and pulse reshaping, can be observed experimentally as an extended channel of plasma.

In addition, the self-regulating mechanism plays a key role in harmonic phase-matching. Despite the long generation medium, nonlinear propagation of the driving pulse reduces the influence of intensity and phase variations to the harmonic phase mismatch, thus geometric and intrinsic contributions can be neglected. Therefore, in our SIGC geometry, the phase mismatch, ∆*k*_*q*_, is mainly due to free electrons and neutral atoms near the laser focus, a situation that resembles that encountered in capillaries^39,40^, or in the high-pressure and short-medium regime^25,26^. Since these two contributions to ∆*k*_*q*_ have opposite signs and vary with the degree of ionization along the driving pulse, perfect phase matching (∆*k*_*q*_ = 0) is only achieved during a finite temporal window^20,21^. If this window is reduced to a single laser half-cycle, a single attosecond pulse can be isolated and, consequently, the XUV spectrum manifests continuous features. Under these circumstances, the phase mismatch of the *q*-th-order harmonic along the propagation direction (*z*) can be approximated by ref.^19^:

1 $$\varDelta {k}_{q}(z,t)\approx P\cdot q\{(1-\eta (z,t))\cdot \delta {n}_{q}\cdot (\frac{2\pi }{{\lambda }_{L}})-\eta (z,t)\cdot {N}_{{atm}}\cdot {r}_{e}\cdot {\lambda }_{L}\}$$

where *P* is the gas pressure, *η*(*z,t*) is the instantaneous ionization fraction, *δn*_*q*_ = *n*(*λ*_*L*_) − *n*(*λ*_*q*_) ~ *n*(*λ*_*L*_) − 1 is the difference between the index of refraction at the fundamental and harmonic wavelengths at a pressure of 1 atm, *N*_atm_ is the total number density of atoms at 1 atm, r_*e*_ represents the classical electron radius, and *λ*_*L*_ is the central wavelength of the driving laser (here assumed *λ*_*L*_ = 700 nm, which is roughly the center of the spectra shown in Fig. 4a).

Finally, the width ∆*t* of the temporal phase-matching window can then be estimated from the inverse of the time derivative of the phase mismatch 1*/*∆*t* ∝ (*∂*∆*k*_*q*_*/∂t*)·*L*, where *L* is the medium length^20^. Note that, under these assumptions, the nonlinear propagation of the driving pulse modifies the role of pressure in harmonic phase-matching. Indeed, while the perfect phase-matching condition (∆*k*_*q*_ = 0, see Eq. (1)) does not depend on pressure, the temporal phase matching window, instead, is inversely proportional to it, ∆*t* ∝ 1*/P*. Therefore, as the pressure increases, the temporal-phase matching window narrows. Note that the increase in harmonic efficiency with pressure is not only limited by the temporal phase-matching window, but also by reabsorption^24^.

**Fig. 5:**
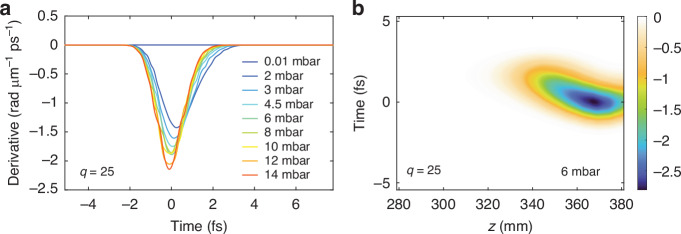
Harmonic phase-matching. **a** Time-derivative of the numerical phase mismatch calculated using Eq.(1) for harmonic 25th at the cell output (*z* = 380 mm). **b** 2D-representation of the phase mismatch time-derivative for harmonic 25th at 6 mbar as a function of the propagation axis, showing that the isolation of an attosecond pulse takes place in the last 30 mm

To illustrate the role of the phase-matching window in the SIGC configuration, in Fig. 5 we show the analytical estimation of its time derivative for the harmonic of order *q* = 25 ( ≈ 44 eV) at the cell output, for the different pressures used in this work. For simplicity, we have just considered the on-axis temporal dependence of the ionization rate *η*(*z,t*) obtained from the 3D nonlinear propagation simulations. This model confirms that the temporal phase matching window shrinks as the gas pressure increases, perfectly aligning with the formation of a continuous cut-off and the isolation of an IAP. For high pressures (>6 mbar) the phase-matching window continues to narrow, eventually becoming too short to allow for the HHG recombination step to occur within the phase-matched interval. This results in a drastic decrease in the XUV signal at higher pressures, as shown in Fig. 2a.

An equally important insight from the numerical analysis concerns the actual phase-matching length within the SIGC geometry. Figure 5b demonstrates that efficient HHG occurs predominantly in the last 3 cm of the cell (350 mm *<* *z* < 380 mm), roughly corresponding to the driving field Rayleigh range in vacuum. Nonetheless, as shown in Fig. 4d, the nonlinear spatial reshaping leads to a progressive increase in the effective Rayleigh range within the medium, reaching up to 10 cm at high gas pressures. As a result, by varying the pressure in our SIGC geometry, we can investigate different ratios between the Rayleigh range and the length of the generating medium. This approach enables the exploration of various phase-matching regimes as outlined in ref.^24^. Moreover, we note that the shortest phase-matching window (phase matching peak) occurs at *z* = 368 mm ~13 mm before the cell output (8 mm before the geometrical laser focus), in excellent agreement with earlier reports on optimal phase matching in SIGC geometries^30^.

IAP generation is further enhanced by the reabsorption of low-order harmonics (see Figs. 2 and 3) favoured by the 7 mm channel connecting the region at a few-mbar pressure to the evacuated region at 10^−2^ mbar in our SIGC. A COMSOL simulation of the gas distribution (see Supplementary Information) revealed a linear pressure gradient developing within the channel with concomitant re-absorption of lower-order harmonics (under 35 eV) as the pressure is increased; conversely, the higher energy spectrum is mostly unaffected due to the Argon cross-section. As a result, the contrast of the IAP is significantly enhanced, effectively functioning as a high-pass spectral filter. It is important to note that in short gas cells operating at high pressures, sharp gradients are instead suggested as the optimal configuration to avoid significant re-absorption effects and poor phase matching^28^. This imposes a sub-optimal compromise between sharp gradient and high-pass filtering for geometries based on short cells (see Supplementary Information).

In conclusion, we demonstrated, both experimentally and theoretically, the feasibility of a SIGC to efficiently generate isolated attosecond pulses in the extreme ultraviolet spectral window. We proved that nonlinear propagation effects of the driving field have a key role in achieving favourable phase-matching conditions. We showed how IAPs can be obtained by only increasing the generation pressure, keeping at the same time a good level of flux. This is attributed to an interplay between an efficient time-gated phase matching and a controlled reabsorption of low-order harmonics.

In order to identify the physics beyond the efficient IAP generation in a SIGC configuration, we have introduced and tested a novel simulation method that combines, for the first time to the best of our knowledge, the nonlinear spatiotemporal reshaping of an intense few-cycle driving field due to propagation through the generation medium with macroscopic HHG comprising full-quantum 3D-TDSE calculations and propagation of the harmonics to a far-field detector. Our simulations and models emphasize that the progressive stagnation of beam size, induced by the spatiotemporal reshaping of the driving field and by plasma defocusing, seen in the experiment as a bright plasma channel, causes the phase-matching in our SIGC geometry to resemble the one encountered in capillaries or in short medium. Consequently, with increasing pressures, the phase matching for isolated attosecond pulses exhibits a straightforward pattern. Due to the balancing of phase mismatch between free electrons and neutral atoms, as the gas pressure increases, the temporal window for phase-matching becomes narrower, thereby enabling the efficient creation of IAPs.

Unlike standard gas cell geometries or capillaries, where scaling up the energy implies modification of the cell length or capillary size^41^, any construction limits do not constrain the coherence length of generation in a SIGC. Consequently, when changing the laser pulse parameters together with the focusing optics, the SIGC region of phase-matching will automatically adapt, requiring only fine adjustment on the gas pressure. This might make the SIGC a very versatile option when scaling up the driving pulse energy, an important issue in view of the growth of high-flux, long geometries beamlines^23,24^. These facts, in addition to its intrinsic simplicity and pressure/generation position tunability, makes the SIGC an attractive candidate for expanding the practical usability of IAP technology in various research fields, e.g., XUV-XUV pump-probe or NIR/VIS/UV pump-XUV probe experiments.

## Materials and methods

### Experimental setup

Our experimental setup uses a 25 fs, 800 nm, 1 kHz, CEP-stabilized (*<*300 mrad) Ti:Sa laser system (Legend DUO HE + , Coherent). These pulses are focused into a 3 m-long stretchable hollow core fiber with a 450 *µ*m inner diameter (Polymicro TSP450670), which is filled with 1.8 bar of helium in a differential gradient configuration. At the output of the fiber, the spectrally broadened IR pulse undergoes post-compression using chirped mirrors (Ultrafast Innovations PC1332), resulting in a sub-4-fs pulse with an energy of 2 mJ.

The laser is then directed into a delay-stabilized interferometer designed for pump-probe experiments, and after a 80/20 beamsplitter and several reflections, 420 *µ*J of pulse energy are focused (*f* = 75 cm) for HHG in the SIGC apparatus. We characterize the IR driving pulse for HHG both in the temporal and spatial domain. The former is performed is by employing the D-scan technique^41^, revealing a temporal duration of 4 fs. The spatial characterization is done by measuring in air the caustic of a small portion of the beam after the focusing element, obtaining a beam waist *w*_0_ = 77 *µ*m and a Rayleigh length *z*_*R*_ = 2.5 cm.

The generated XUV radiation passes through a 150 nm Al filter to remove the remaining portion of the driving IR field and filter out the harmonics below 20 eV. The XUV radiation is then first focused by a gold toroidal mirror into a time-of-flight spectrometer (TOF, Kaesdorf ETF15), and recombined with sub-4-fs IR pulses to perform streaking (FROG-CRAB) measurements^42^. The pump-probe delay is actively stabilized with an rms below 50 mrad. The XUV radiation is collected by a gold-coated grating (Hitachi 600 lines/mm), where it is spectrally dispersed and directed towards a microchannel plate (MCP) for measuring the radiation spectrum using a phosphor screen and a camera.

### Theoretical simulations

The complete theoretical description of HHG requires to couple the propagation of the driving field with the generation of the high-order harmonics at the single-atom level and their subsequent propagation. However, such calculation is tremendously expensive computationally. First, at the microscopic level, the exact laser-driven dynamics of the quantum electronic wavepacket in the HHG process is described by the 3D-TDSE. The full 3D-TDSE calculation could include the dynamics of all electrons in the atom but, as the main physics of HHG is accurately described by the electron occupying the outermost valence orbital, we work under the commonly used single-active electron approximation. Second, at the macroscopic level, the emission from all atoms in the gas cell—typically trillions—must be considered to account for harmonic phase-matching, together with the propagation of the driving field. Several methods have been successfully developed to account for macroscopic HHG, using the 3D-TDSE^20,36,43–45^, the strong field approximation^46,47^, which cannot be used when dealing with few-cycle drivers^48^, or even artificial intelligence^49^. Although separate simulations of nonlinear propagation of the driving field and HHG have already been performed, for example, to successfully account for soft X-ray generation in a waveguide through HHG^39^, to the best of our knowledge there is no previous method that couples nonlinear few-cycle IR propagation with macroscopic HHG that makes use of the 3D-TDSE at the single-atom level.

In our simulations we employ the 3D-TDSE in Argon under the single-active electron approximation. The resulting harmonics are then propagated to a far-field detector through the electromagnetic field propagator, and phase-matching is considered by coherently adding the elementary contributions along the gas cell, following the method described in ref.^47^. For each emitter location, the associated IR driving field was computed with a 3D nonlinear pulse propagation code^50^, which takes the experimental parameters as an input and solves the IR propagation in the complete SIGC volume. These propagation simulations for the IR driver included the complete dispersion of the gas, the Kerr effect, self-steepening and shock terms, and photoionization and plasma absorption. To further strengthen our investigation, we used a realistic gas profile distribution, calculated using computational fluid dynamics tools (COMSOL). This gas profile was included in the propagation of the harmonic radiation through the 200 *µ*m-diameter and 7-mm-long channel connecting the first two chambers of the SIGC (here, further generation was neglected), while the gas pressure in the HHG generation volume was assumed to be constant.

In order to realistically simulate the nonlinear propagation in the SIGC and the HHG process, the results of the experimental temporal and spatial characterizations (4 fs pulses, beam waist *w*_0_ = 77 *µ*m and Rayleigh length *z*_*R*_ = 2.5 cm) were used as the input for the nonlinear propagation simulations.

### Supplementary information

Supplementary material

## Data Availability

The data that support the findings of this study are available from the corresponding authors upon reasonable request.
