# Supplementary material for: Isolated attosecond pulse generation in a semi-infinite gas cell driven by time-gated phase matching
Source: Light Sci Appl. 2024 Aug 20;13:197. doi: 10.1038/s41377-024-01564-5 (PMC11336177; doi:10.1038/s41377-024-01564-5)
Supplement: Supplementary file 1 — Supplementary material [file 41377_2024_1564_MOESM1_ESM.docx]

**Supplementary Information:**

**Isolated attosecond pulse generation in a semi-infinite gas cell driven by time-gated phase matching**

Federico Vismarra,^1,2,+^ Marina Fernández-Galán,^3,4,+^ Daniele Mocci,^1,+^ Lorenzo Colaizzi,^1^ Víctor Wilfried Segundo,^3,4^ Roberto Boyero-García,^3^ Javier Serrano,^3,4^ Enrique Conejero-Jarque,^3,4^ Marta Pini,^1,2^ Lorenzo Mai,^1^ Yingxuan Wu,^1^ Hans Jakob Wörner,^5^ Elisa Appi,^6^ Cord L. Arnold,^6^ Maurizio Reduzzi,^1^ Matteo Lucchini,^1,2^ Julio San Román,^3,4^ Mauro Nisoli,^1,2,*^ Carlos Hernández-García,^3,4,*^ and Rocío Borrego-Varillas^2,*^

^1)^ *Department of Physics, Politecnico di Milano, Piazza Leonardo da Vinci 32, 20133 Milano (Italy)*

^2)^ *IFN-CNR, Piazza Leonardo da Vinci 32, 20133 Milano (Italy)*

^3)^ *Grupo de Investigación en Aplicaciones del Láser y Fotónica, Departamento de Física Aplicada, Universidad de Salamanca, E-37008 Salamanca (Spain)*

^4)^ *Unidad de Excelencia en Luz y Materia Estructuradas (LUMES), Universidad de Salamanca, Salamanca (Spain)*

^5)^ *Laboratorium für Physikalische Chemie, ETH Zürich, 8093 Zürich (Switzerland)*

^6)^ *Department of Physics, Lund University, Lun, (Sweden)*

I. **GAS PROFILE STUDY AND RE-ABSORPTION OF LOW-ORDER HARMONICS**

To gain deeper insights into the physical mechanism behind the low-order harmonic reabsorption process, discussed in the main text, we conducted a detailed analysis of the gas distribution in our generation geometry, computed in COMSOL. As mentioned in the main text, the SIGC was designed with a 7-mm channel connecting the region at a few-mbar pressure to the evacuated region at 10^−2^ mbar. Under the assumption that the generation in this intermediate region is negligible, we can calculate the total transmittivity *T*(*ε*) across this channel as a function of photon energy using Eq. (1).

$T(\varepsilon) = exp\left( -\frac{L}{L_{abs}}\left( \varepsilon\right) \right); L_{abs}(\varepsilon) = \int_{0}^{7 mm} \frac{dz}{(\rho(z)\sigma_{abs}(\varepsilon))}$ **(1)**

where *ρ*(*z*) is the atomic areal density of the medium as a function of *z*, while *σ_abs_*(*ε*) is the absorption cross-section of Argon^1^. As presented in Fig. S1(a), the spectral shape of the transmittivity perfectly explains the re-absorption feature in our experimental XUV spectrum. With increasing pressure, the low-order harmonics (*<* 35 eV) undergo significant re-absorption, while higher energies are preserved due to the shape of Argon’s cross-section, resulting in almost 100% transmittivity.


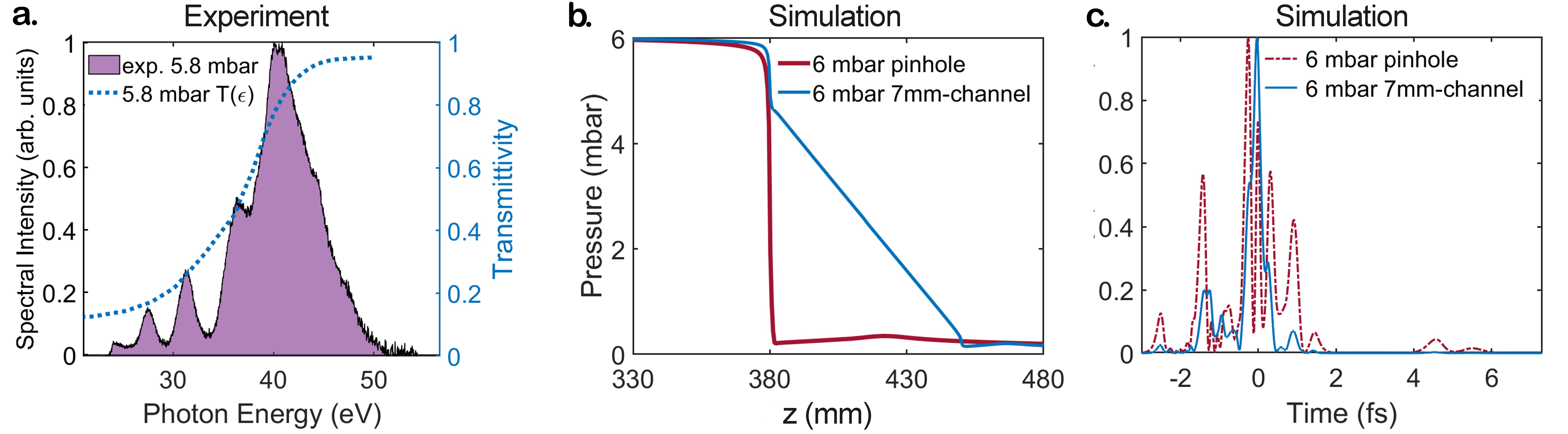


FIG. S1. (a) Experimental (purple area) spectrum obtained at a pressure of 6 mbar. The dotted blue line indicates the calculated *z*-integrated transmittivity in the 7-mm channel of the SIGC. (b) Comparison between the gas distribution of a 7-mm channel and a geometry with a pinhole, i.e., immediate connection to vacuum. (c) Numerical results of XUV temporal profile in the two configurations of panel (b).

It is essential to highlight that this simplicity in managing the gas profile through a channel is easily achievable in geometries operating at low generation pressure, such as the SIGC. By simply enabling the gas to flow through a few millimeters channel, we generate a linear gradient of pressure, illustrated by the blue curve in Fig. S1(b). This gas channel serves as a high-pass filter, selectively transmitting the desired spectral region. It is important to emphasize that within the channel, where harmonic generation can be neglected, the specific shape of the gas profile is

irrelevant, as what matters for absorption is its integrated contribution.

Conversely, in shorter gas cells operating at much higher pressures, achieving the same level of control becomes challenging. Indeed, especially at high pressure, sharp gradients are suggested as the optimal configuration to avoid significant re-absorption effects across the whole spectrum and poor phase matching^2^, making necessary a sub-optimal compromise between sharp gradient and high-pass filtering. To further stress the key role played by this channel, we performed an additional HHG simulation assuming a sharp edge in the gas profile (red curve in Fig. S1(b)), mimicking a pinhole directly interfaced with the evacuated chamber. As shown in Fig. S1(c), the temporal quality of the generated XUV radiation is dramatically reduced with respect to the case of a linear gradient, in which, as already presented, a single attosecond pulse can be isolated (blue curve in Fig. 1(c)). We estimated the optimal length in our configuration to keep the transmittivity

in the 40 eV region above 90% and granting at the same time good filtering of the low-order harmonics to be between 1 cm - 1*.*25 cm. Of course, depending on the channel diameter, its length should be adapted. A full investigation of this matter is beyond the scope of this work.

**2. CEP DEPENDENT XUV SPECTRA**


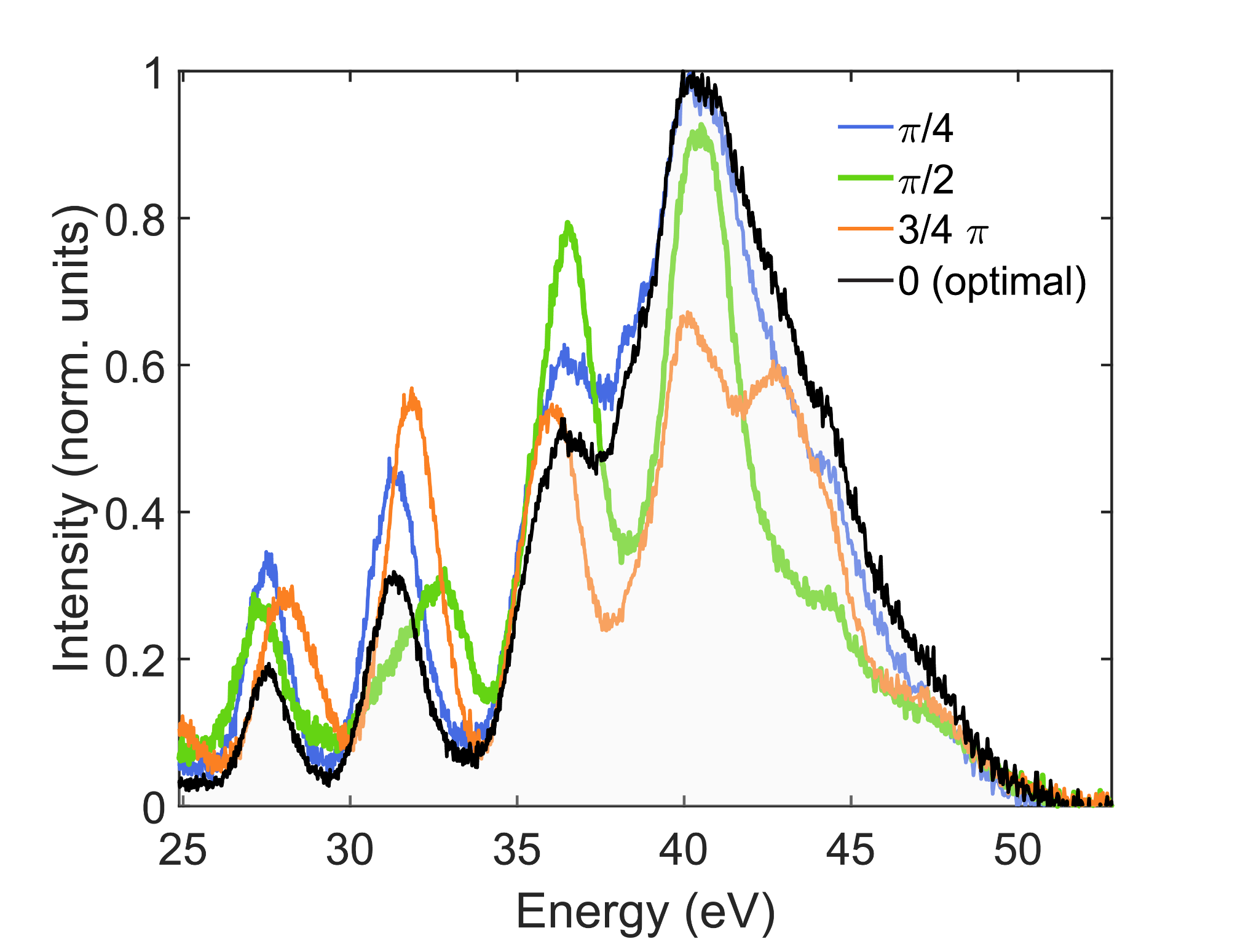


FIG. S2. XUV spectra recorded under the experimental conditions reported in the main text for different values of the CEP

**REFERENCES**

^1^B. Henke, E. Gullikson, and J. Davis, “X-Ray Interactions: Photoabsorption, Scattering,

Transmission, and Reflection at E = 50-30,000 eV, Z = 1-92,” Atomic Data and Nuclear Data Tables **54**, 181–342 (1993).

^2^B. Major and K. Varjú, “Extended model for optimizing high-order harmonic generation in absorbing gases,” Journal of Physics B: Atomic, Molecular and Optical Physics **54**, 224002 (2022).
